# From Misdiagnosis to Genetic Confirmation: A Brazilian Familial Report of Camptodactyly–Arthropathy–Coxa Vara–Pericarditis Syndrome—A Case‐Based Review

**DOI:** 10.1155/crpe/8908027

**Published:** 2026-01-28

**Authors:** Ana Luiza Garcia Cunha, Isabela Tavares Barretos Matias, Matheus Santos França, Joziele de Souza Lima

**Affiliations:** ^1^ Pediatric Rheumatology Department, Hospital Infantil Joao Paulo II, Belo Horizonte, Minas Gerais, Brazil; ^2^ Pediatric Department, Hospital Infantil Joao Paulo II, Belo Horizonte, Minas Gerais, Brazil; ^3^ Genetics Department, Hospital Infantil Joao Paulo II, Belo Horizonte, Minas Gerais, Brazil

**Keywords:** camptodactyly–arthropathy–coxa vara–pericarditis syndrome, case report, juvenile idiopathic arthritis, trigger finger, whole-genome sequencing

## Abstract

**Background:**

Camptodactyly–arthropathy–coxa vara–pericarditis (CACP) syndrome is a rare autosomal recessive disorder caused by *PRG4* mutations that impair lubricin production. Resulting noninflammatory hyperplasia produces congenital or early‐onset camptodactyly and noninflammatory arthropathy, affecting large joints. Because clinical features overlap with trigger finger and juvenile idiopathic arthritis (JIA), misdiagnosis is common.

**Case Presentation:**

We describe the second genetically confirmed Brazilian case of CACP, involving two siblings. Both showed congenital trigger fingers (later reclassified as camptodactyly) and developed painless, cold swelling of large joints, initially labeled JIA. Laboratory tests showed normal inflammatory markers, and synovial fluid revealed low white cell counts. Imaging demonstrated joint effusion and synovial debris without inflammatory signs. Whole‐genome sequencing identified a homozygous c.3756dup mutation in the *PRG4* gene, introducing a premature stop codon and truncating lubricin.

**Conclusion:**

This report highlights the importance of recognizing CACP syndrome by identifying distinctive clinical, laboratory, and imaging characteristics, notably congenital camptodactyly and noninflammatory joint swelling, to prevent misdiagnosis and guide supportive management.

## 1. Introduction

Camptodactyly–arthropathy–coxa vara–pericarditis syndrome (CACP; OMIM 208250) is a rare autosomal recessive disease caused by mutations in the proteoglycan 4 (*PRG4*) gene [1–3]. The noninflammatory synovial hyperplasia results in congenital or early‐onset camptodactyly and early‐onset noninflammatory arthropathy, primarily involving large joints such as the knees, ankles, elbows, and hips. Additionally, it may progressively lead to bilateral coxa vara deformity and sterile pericarditis [4, 5]. Some cases also present with the involvement of other joints, such as the temporomandibular joint, suggesting a phenotypic variability of CACP syndrome [6, 7]. Due to the characteristic chronic joint swelling, some cases are mistakenly diagnosed as juvenile idiopathic arthritis (JIA), with the correct diagnosis being made later [7–10]. Differentiation between CACP syndrome and JIA is important since treatment and prognosis differ significantly.

Noninflammatory pericarditis has been reported in approximately 33% of published CACP cases. Pericarditis is usually mild and self‐limiting, but in rare cases, it can be severe. Coxa vara has been observed in 50%–100% of CACP syndrome cases [3, 11].

The incidence and prevalence of CACP syndrome are unknown; however, it is more common in populations with high consanguinity rates, such as in Saudi Arabia, the United Arab Emirates, and Egypt [12, 13]. Nonetheless, cases have been reported in various populations worldwide, indicating that CACP syndrome may be underdiagnosed in other regions [7, 8].

The disease gene *PRG4* has been mapped to human chromosome 1q25‐q31, where mutations result in a prematurely truncated protein [9].

*PRG4* is a glycosylated, O‐linked, secreted megakaryocyte‐stimulating protein synthesized by chondrocytes located at the surface of articular cartilage and by some synovial lining cells. The pathogenesis of CACP syndrome has been associated with defective *PRG4*, also known as lubricin, found in synovial fluid, on the surface of cartilage, and in tendons. In addition to its lubricating function, lubricin regulates synovial growth [4, 5, 9, 14].

Recently, a case was reported of a 33‐year‐old Brazilian patient with the same *PRG4* mutation (c.3756dup (p.Lys1253Ter)) as identified in our patients, marking the first genetically confirmed case of CACP syndrome in Brazil [15]. This article presents two additional cases involving siblings with the same mutation, representing Brazil’s second genetically confirmed case. Despite residing in different regions of Brazil and not being genetically related, these patients share the same genetic variant, which has not been previously described phenotypically in pediatric patients or described in other worldwide regions.

## 2. Case Presentation

### 2.1. Patient 1

**FIGURE 1:**
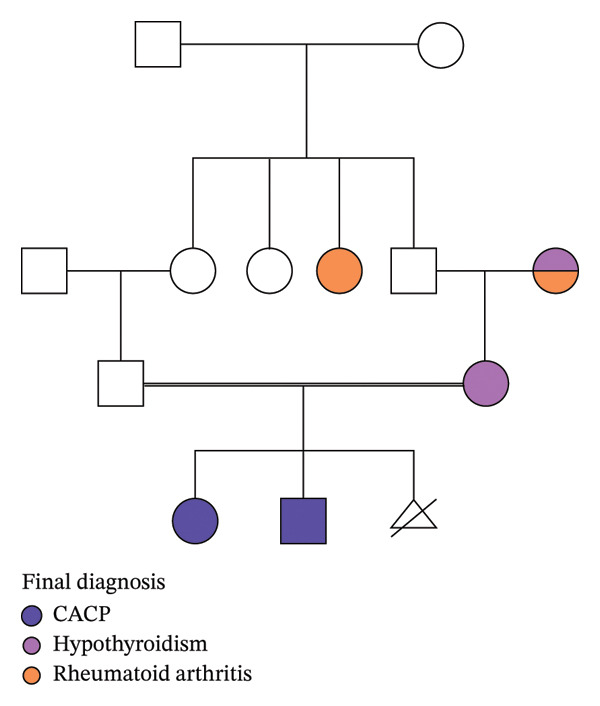
Pedigree chart.

Patient 1 is a 7‐year‐old male. At birth, he presented with bilateral finger movement limitations and was diagnosed by orthopedics with multiple trigger fingers involving all fingers on both hands. He was also born with a sebaceous nevus of Jadassohn on his scalp, which was surgically removed. At 10 months old, he was referred to pediatric rheumatology with suspected JIA due to persistent trigger fingers. At that time, no inflammatory joint involvement was noted, and a geneticist evaluation was recommended due to parents’ consanguinity (Figure 1) and the presence of congenital trigger fingers, which were also observed in his sister. However, the patient was not evaluated by a geneticist then and underwent surgical correction of the trigger fingers.

At age 3, he developed bilateral knee swelling and was referred again for clinical reevaluation of JIA. He presented with cold, voluminous swelling in both knees and ankles, thickening in both elbows and wrists, and flexion of all fingers. Notably, he did not have significant pain with active or passive joint movement, and his laboratory tests were normal, with no leukocytosis and negative inflammatory markers.

**FIGURE 2:**
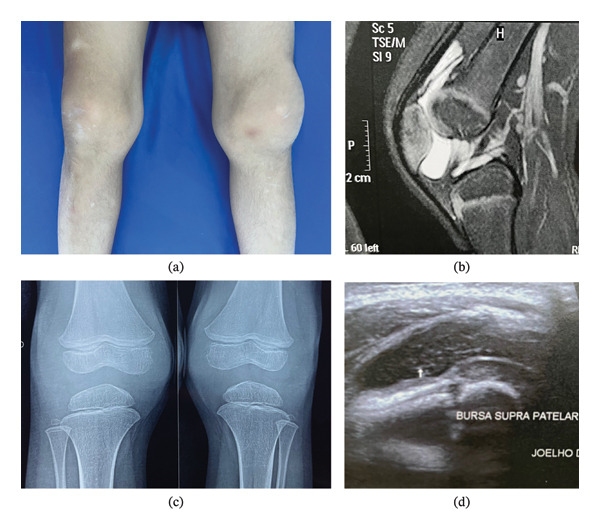
Patient 1: (a) voluminous bilateral knee swelling; (b) MRI of the right knee showing moderate joint effusion; (c) X‐ray showing increased joint space in the knees; (d) knee ultrasound showing debris in the suprapatellar bursa.

Further investigation included a knee ultrasound, which revealed anechoic images with internal debris in the suprapatellar bursa and the medial and lateral joint recesses (Figure 2). Synovial fluid aspiration from the left knee showed a low white cell count (45 white blood cells per mm^3^, 48% macrophages, 8% neutrophils, 5% lymphocytes, and 39% mesothelial mononuclear and multinucleated cells). Knee MRI showed moderate joint effusion and thickened suprapatellar and infrapatellar plicae but otherwise normal findings. His echocardiogram was normal.

At age 5, an intra‐articular triamcinolone injection was administered to the knees due to localized pain, with transient improvement in swelling but no complete resolution and recurrence within six months. Lysosomal storage diseases were hypothesized due to the presence of crystals in the synovial fluid, the absence of systemic inflammation, and a poor response to intra‐articular steroid injections. At the end of that year, genetic sequencing was requested for the patient and his sister. While awaiting genome sequencing results, a trial of methotrexate was initiated for 4 months, with no change in the clinical course. After receiving the genetic sequencing results (described below), methotrexate was discontinued, and counseling regarding the lack of specific treatment was provided, alongside intensified multidisciplinary management with physical and occupational therapy.

### 2.2. Patient 2

**FIGURE 3:**
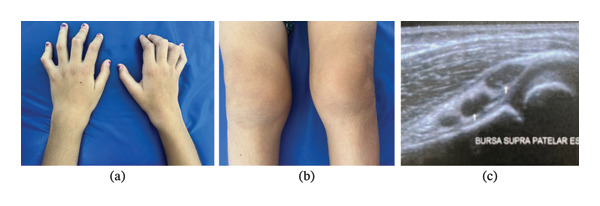
Patient 2: (a) camptodactyly with bilateral finger flexion and bilateral wrist swelling; (b) bilateral knee swelling; (c) knee ultrasound showing debris in the suprapatellar bursa.

The second patient, a 9‐year‐old female (Figure 3), was born with trigger thumbs, which were surgically corrected. During her early years, she was followed by pediatric orthopedics due to suspected congenital clubfoot, which was clinically ruled out. At age 5, when her brother was evaluated at 3 years old, she was also found to have cold, painless swelling in the knees, ankles, and wrists, similar to her brother’s physical findings, as well as flexion positioning of her fingers. Like her brother, she had normal laboratory tests without systemic inflammation, and a bilateral knee ultrasound revealed debris in the synovial fluid. Over the past 3 years, she has experienced worsening finger flexion while maintaining swelling in large joints.

She was also treated with methotrexate for 4 months without clinical improvement. After her brother’s genetic sequencing results, methotrexate was discontinued, and counseling was provided regarding the current lack of specific treatment, along with intensified multidisciplinary management, including physical and occupational therapy.

### 2.3. Genetic Sequencing

Whole‐genome sequencing was performed on both patients, and molecular methods have been described elsewhere [16], revealing a pathogenic mutation previously described in genetic databases but without phenotypic description in pediatric patients until now. The c.3756dup (p.Lys1253Ter) variant was found in both siblings, in homozygosity, in the *PRG4* gene (NM_M_005807.6), Exon 10, and associated with CACP syndrome. This duplication creates a premature stop codon at amino acid 1253, leading to a truncated lubricin protein and disrupting synovial growth regulation.

### 2.4. Ethical Approval

The institutional ethics committee approved this case report and the genetic sequencing project. The genetic sequencing project received approval under CAAE: 29567220.4.2021.5119, approval number: 6.982.378, and the case report was approved under CAAE: 79507324.9.0000.5119, approval number: 6.815.508. Written informed consent was obtained from the parents to publish clinical data and images.

## 3. Discussion

To date, 66 pathogenic/probably pathogenic variants have been described in the *PRG4* gene most of them associated with CACP syndrome [17]. Most of the patients described are of Arab origin. Previously, the first genetically confirmed case in Brazil involved a 33‐year‐old male with the same *PRG4* mutation (c.3756dup (p.Lys1253Ter)) [15]. Our report presents the second genetically confirmed case in Latin America, involving two siblings from a geographically different Brazilian region than the first reported case but sharing the same mutation. This suggests a possible founder effect or underrecognized prevalence in the Brazilian population.

CACP syndrome is characterized primarily by the association of congenital or early‐onset camptodactyly and noninflammatory arthropathy with synovial hyperplasia. These characteristics can mimic more common conditions.

In this case, two important differential diagnoses were considered before the final diagnosis was established. Initially, both patients were diagnosed and surgically treated in early childhood for trigger fingers. These patients likely exhibited congenital camptodactyly, making the diagnosis of trigger finger inappropriate. The majority of patients with congenital trigger fingers have involvement of the thumbs, with 54% presenting unilaterally. However, approximately 30% of cases are secondary to genetic causes, particularly mucopolysaccharidoses and related lysosomal storage diseases, making geneticist evaluation essential [8]. Genetic causes are more often associated with multiple bilateral trigger fingers and carpal tunnel syndrome. Additionally, primary trigger finger rarely recurs (6%) after surgical correction, unlike in the described cases, where both patients experienced worsening after surgery [18].

The second differential diagnosis raised was JIA, leading to the patient’s follow‐up with pediatric rheumatology. CACP syndrome can easily be mistaken for JIA, especially the polyarticular form, due to chronic joint swelling. However, certain features help distinguish between them. While JIA often presents with systemic inflammatory signs, such as elevated inflammatory markers and significant joint pain, in CACP syndrome, inflammatory markers are typically normal, and joint pain is less prominent [7, 9]. Furthermore, patients with CACP syndrome often do not respond to immunosuppressive therapies used in JIA, as observed in the cases reported in the literature [8, 10].

From the beginning, atypical features for polyarticular JIA were noted, such as low inflammatory markers, cold joint swelling, and the ultrasound findings of intra‐articular debris in the knees of both patients, along with low inflammatory cell counts in the synovial fluid. These findings align with the previously described characteristics of CACP syndrome, where large joint effusions are typically noninflammatory and present variable echogenicity—anechoic, hypoechoic, or mixed—on ultrasound examination. Similarly, our patients exhibited abundant synovial fluid with fine, mobile hyperechoic echoes, lacking posterior acoustic shadowing, which created a speckled ultrasound pattern suggestive of proteinaceous material, cellular debris, or microcrystals. Such findings, when encountered early in the disease course, strongly support the consideration of CACP syndrome in the appropriate clinical context [19].

Despite a high suspicion of an underlying genetic condition, Patient 1 underwent intra‐articular triamcinolone injections, and both patients were treated with methotrexate for 4 months without a significant clinical response.

When considering differential diagnoses, pediatricians, orthopedists, and rheumatologists need to recognize phenotypic features such as camptodactyly, finger flexion, and cold, voluminous joint swelling. Prompt referral for genetic testing can prevent the potential harm of immunosuppressive therapies and invasive procedures in these cases. Moreover, appropriate management of CACP syndrome primarily involves supportive measures, such as physical and occupational therapy and, in some cases, orthopedic interventions to correct deformities. Currently, no specific therapies are available, highlighting the need for early and accurate diagnosis [6, 10].

Generally, the disease prognosis is good, with preservation of articular function with supportive measures. However, pericarditis can occur during follow‐up, and it is important to monitor for cardiovascular symptoms; rarely, tenosynovectomy is necessary to relieve articular symptoms.

This report documents the second genetically confirmed case of CACP syndrome in Brazil—a rare, underdiagnosed condition—and provides the first detailed phenotypic description of the *PRG4* gene mutation c.3756dup (p.Lys1253Ter) in pediatric patients. Despite residing in different Brazilian states, the patients share the same genetic mutation, emphasizing the need for increased awareness among clinicians.

CACP syndrome can easily be mistaken for JIA, mainly due to the overlap of chronic joint swelling. However, the distinct characteristics of CACP, including noninflammatory joint effusions, cold swelling, and camptodactyly, should prompt clinicians to consider genetic testing to avoid misdiagnosis and inappropriate treatments. Management should focus on supportive care, including physical and occupational therapy, as there is currently no specific treatment for CACP syndrome.

## Funding

No funding was received for this manuscript.

## Conflicts of Interest

The authors declare no conflicts of interest.

## Data Availability

The data that support the findings of this study are available upon request from the corresponding author. The data are not publicly available due to privacy or ethical restrictions.

## References

[1] Bahabri S. A. , Suwairi W. M. , Laxer R. M. , Polinkovsky A. , Dalaan A. A. , and Warman M. L. , The camptodactyly-arthropathy-coxa vara-pericarditis Syndrome: Clinical Features and Genetic Mapping to Human Chromosome 1, Arthritis & Rheumatism. (1998) 41, no. 4, 730–735. 9550484 10.1002/1529-0131(199804)41:4<730::AID-ART22>3.0.CO;2-Y

[2] Marcelino J. , Carpten J. D. , Suwairi W. M. et al., CACP, Encoding a Secreted Proteoglycan, is Mutated in camptodactyly-arthropathy-coxa vara-pericarditis Syndrome, Nature Genetics. (1999) 23, no. 3, 319–322, 10.1038/15496, 2-s2.0-0032699840. 10545950

[3] Yilmaz S. , Uludağ Alkaya D. , Kasapçopur Ö. et al., Genotype-Phenotype Investigation of 35 Patients from 11 Unrelated Families with camptodactyly-arthropathy-coxa vara-pericarditis (CACP) Syndrome, Molecular Genetics Genomic Medicine. (2018) 6, no. 2, 230–248, 10.1002/mgg3.364, 2-s2.0-85045522990. 29397575 PMC5902402

[4] Martínez-Lavín M. , Buendía A. , Delgado E. et al., A Familial Syndrome of Pericarditis, Arthritis, and Camptodactyly, New England Journal of Medicine. (1983) 309, no. 4, 224–225, 10.1056/nejm198307283090407, 2-s2.0-0020957250. 6866038

[5] Bulutlar G. , Yazici H. , Ozdogan H. , and Schreuder I. , A Familial Syndrome of Pericarditis, Arthritis, Camptodactyly, and Coxa Vara, Arthritis & Rheumatism. (1986) 29, no. 3, 436–438, 10.1002/art.1780290321, 2-s2.0-0022656722. 3964320

[6] Maniscalco V. , Pizzetti C. , Marrani E. et al., Pseudo-Rheumatic Manifestations of Limping: Camptodactyly-Arthropathy-Coxa vara-pericarditis Syndrome—Single Case Report and Review of the Literature, Frontiers in Pediatrics. (2022) 10, 10.3389/fped.2022.981938. PMC9760854 36545657

[7] Kakkar R. M. , Soneji S. , Badhe R. R. , and Desai S. B. , Camptodactyly-Arthropathy-Coxa vara-pericarditis Syndrome: Important Differential for Juvenile Idiopathic Arthritis, Journal of Clinical Imaging Science. (2013) 3, 10.4103/2156-7514.114211, 2-s2.0-84940231608. PMC3779395 24083061

[8] Johnson N. , Chaudhary H. , Kumrah R. et al., Syndrome of Progressive Deforming Non-inflammatory Arthritis of Childhood: Two Patients of camptodactyly-arthropathy-coxa vara-pericarditis Syndrome, Rheumatology International. (2021) 41, no. 10, 1875–1882, 10.1007/s00296-020-04688-0. 32813152

[9] Bağrul İ. , Ceylaner S. , Yildiz Y. T. et al., A Novel Mutation in the Proteoglycan 4 Gene Causing CACP Syndrome: Two Sisters Report, Pediatrics Rheumatology Online Journal. (2023) 21, no. 1, 10.1186/s12969-023-00793-z. PMC9875468 36694203

[10] Kisla Ekinci R. M. , Balci S. , Dogan H. et al., Camptodactyly-Arthropathy-Coxa Vara-Pericarditis Syndrome Resembling Juvenile Idiopathic Arthritis: a single-center Experience from Southern Turkey, Molecular Syndromology. (2021) 12, no. 2, 112–117, 10.1159/000513111. 34012381 PMC8114071

[11] Peters B. , Schuurs-Hoeijmakers J. H. , Fuijkschot J. et al., Protein-Losing Enteropathy in camptodactyly-arthropathy-coxa vara-pericarditis (CACP) Syndrome, Pediatrics Rheumatology Online Journal. (2016) 14, no. 1, 10.1186/s12969-016-0093-5, 2-s2.0-84969963105. PMC4880819 27224999

[12] Alazami A. M. , Al-Mayouf S. M. , Wyngaard C. A. , and Meyer B. , Novel *PRG4* Mutations Underlie CACP in Saudi Families, Human Mutation. (2006) 27, no. 2, 10.1002/humu.9399, 2-s2.0-33745939347. 16429407

[13] Offiah A. C. , Woo P. , Prieur A. M. , Hasson N. , and Hall C. M. , Camptodactyly-Arthropathy-Coxa vara-pericarditis Syndrome Versus Juvenile Idiopathic Arthropathy, American Journal of Roentgenology. (2005) 185, no. 2, 522–529, 10.2214/ajr.185.2.01850522, 2-s2.0-25644449686. 16037531

[14] Krakow D. , Rimoin D. L. , Pyeritz R. E. , and Korf B. R. , The Dysostoses, Emery and Rimoin’s Principles and Practice of Medical Genetics, 2013, 6th edition, Academic Press, 1–22.

[15] Donis K. C. , Kalil M. A. B. , Poswar F. et al., An Adult with Cystathionine beta-synthase Deficiency, camptodactyly-arthropathy-coxa vara-pericarditis Syndrome, and Deafness: a Case Report, Genetics and Molecular Biology. (2024) 47, no. 1, 10.1590/1678-4685-gmb-2022-0335. PMC11003653 38593426

[16] Coelho A. V. C. , Mascaro-Cordeiro B. , Lucon D. R. et al., The Brazilian Rare Genomes Project: Validation of Whole Genome Sequencing for Rare Diseases Diagnosis, Frontiers in Molecular Biosciences. (2022) 9, 10.3389/fmolb.2022.821582. PMC9108541 35586190

[17] ClinVar Database. National Center for Biotechnology Information (NCBI), 2025, https://www.ncbi.nlm.nih.gov/clinvar/.

[18] Wong A. L. , Wong M. J. , Parker R. , and Wheelock M. E. , Presentation and Aetiology of Paediatric Trigger Finger: a Systematic Review, Journal of Hand Surgery European. (2022) 47, no. 2, 192–196, 10.1177/17531934211035642. PMC8873964 34610771

[19] Sparchez M. and Fodor D. , Ultrasound’s Role in Differentiating camptodactyly-arthropathy-coxa vara-pericarditis (CACP) Syndrome from Inflammatory Arthritis in Children. A Narrative Review, Medical Ultrasonography. (2024) 27, no. 4, 10.11152/mu-4452. 39705621

